# ‘The best interest of patients, not self-interest’: how clinicians understand altruism

**DOI:** 10.1186/s12909-021-02908-0

**Published:** 2021-09-07

**Authors:** Madiha Sajjad, Shazia Qayyum, Samina Iltaf, Rehan Ahmed Khan

**Affiliations:** 1 Pathology department, Riphah International University, 274 Peshawar road, Rawalpindi, Pakistan; 2 Surgery department, Riphah International University, 274 Peshawar road, Rawalpindi, Pakistan

**Keywords:** Altruism, Altruistic practices, Professionalism

## Abstract

**Background:**

Altruism refers to acting in ‘the best interest of patients, not self-interest’. With an observed discordance between the concept and practice of altruism, and increasing attention to ‘pathologic altruism’, the role of altruism is blurred in present day medical care. In this background, the required balance of altruistic attitude which needs to be fostered in medical students needs clarity. This problem may be best addressed by the practicing clinicians. The objectives of this study were to explore clinicians’ understanding of altruism in the clinical context and to identify the key concepts of altruism which they felt, must be included in clinical practice.

**Methods:**

It was an exploratory qualitative study to identify clinicians’ understanding of altruism and the key practice points for altruism. Online semi-structured interviews were conducted from 18 clinicians through Zoom and transcribed using Otter. Open coding of interview transcripts was done using Atlas ti 8 and grouped by commonalities under sub themes and themes.

**Results:**

The main concepts regarding the clinicians’ understanding of altruism were prioritizing patients’ interest above oneself, favouring patients beyond routine duty and organized team work for practicing altruism. The essential practice areas identified for altruism were finding a balance between altruistic tendency and self/family life, identifying one’s individual capacity for altruism, establishing teamwork for developing a workplace altruistic attitude, and facilitating patients beyond routine duty.

**Conclusions:**

Altruism is an important professionalism attribute for clinicians, however prioritizing patients interest requires a balanced approach so that it is effective and sustainable. Workplace altruistic cultures may be better promoted through organized team-based approach rather than individual efforts.

**Supplementary Information:**

The online version contains supplementary material available at 10.1186/s12909-021-02908-0.

## Background

Professionalism is a fundamental component of medical profession which shapes the quality of medical care provided by the health care workers. The American Board of Internal Medicine (ABIM), identified the core components of medical professionalism in its ‘Project Professionalism’, one of whose key elements is altruism described as ‘the best interest of patients, not self-interest’ [1]. The Merriam-Webster dictionary defined altruism as an “unselfish regard for or devotion to the welfare of others” [2]. Although, both of these mentioned sources do not necessarily imply its meaning as ‘self-sacrifice’, much of the literature [3–5] alluded to this meaning as a prevalent concept, which brought to it a misguided impression of moral superiority [3] beyond professional obligation [6].

Thus, a dispute emerged, questioning even the retention of altruism as an element of medical professionalism due to reservations about it being too unrealistic and idealistic for practice [4]. At the least, there were suggestions of replacing the term with others like ‘pro-sociality’ or ‘beneficence’, to capture the obligatory nature of a doctor’s relation with the patients in a better manner [4, 7]. Furthermore, the concept of ‘pathological altruism’ emerged in recent literature as altruistic behaviours taken to an extreme, causing adverse rather than beneficial patient outcome and/or work–life imbalance of doctors [6]. More recent studies advocate aligning self-interest with the interest of others termed as ‘enlightened self-interest’, which is of benefit to both the patients as well as the doctors [8]. They contend that it would result in small consistent practices of altruism which are more achievable and sustainable.

Altruistic behaviour may be understood as a complex process accounted for and shaped by multiple factors [4, 9]. These include sociocultural, religious, gender and economic factors, to name a few. According to literature, culture influences altruistic behaviours beyond one’s own community and group level. Religion also effects altruism by influencing the moral values and behaviours [9]. Altruistic tendencies are also affected by economic factors in health care systems. Economically driven health care systems negatively affect altruism [4].

There is still an alleged ambiguity in the understanding of altruism, and an observed discordance between the perceived concept and its clinical practice. Also, there is a lack of agreement on what to consider as the essential components of altruism which need to be taught to our medical students as a must feature of professionalism [8]. This subject is directly linked to everyday clinical practice and may be best gauged by the practicing clinicians. However, there is a lack of clinician perspectives about altruism [7] and the required balance of altruistic attitude which needs to be fostered in medical students, requires clinicians’ input. To bridge this gap in literature, we sought to identify clinicians’ understanding of altruism and their recommendations regarding the essential altruistic behaviors required for medical practice. For this, we devised the following two research questions:

1. What is the understanding of clinicians regarding altruism, based on their experiences?
2. What are the essential altruistic behaviors required to be practiced in the medical field?

## Methodology

It was an exploratory qualitative study conducted at Riphah International University between October and December 2020.

### Research philosophy

#### Epistemological position

The Epistemological stance used in our study was constructivism, in which knowledge is constructed depending on interactions between individuals and their world and develops within a social context [10]. The reasons for this epistemological assumption were the nature of research questions of the study, and the social context of the study. The theoretical perspective, methodology, data collection and analysis were all based on this assumption.

#### Theoretical perspective

As the epistemological position of our study was constructivism, the theoretical perspective informing the methodology was interpretivism [10].

The theoretical/conceptual framework was based on the ‘Enlightened Self-Interest in Altruism (ESIA)’ [8]. This framework attempts to synergize interest of both the doctors and the patients, with the goal of promoting altruistic behaviors in physician and medical students that benefit everyone. Elements contributing to ESIA are: balanced self-sacrifice, personal ambition, altruism, professionalism and desire to help others.

The context of the study was clinicians doing hospital-based practices at various clinical departments in different institutions and involved in teaching undergraduate or postgraduate medical students. This was done to probe their comprehension with regards to teaching requirement of students.

Purposive maximum variation sampling technique was used to select clinicians, with variable ages and work experiences, working in various clinical specialties and institutions. The prospective participants were sent an email invitation to participate along with relevant information to familiarize them with the term ‘altruism’.

There were 18 participants, including 10 females and 8 males from 6 different institutions. The range of clinical specialties included surgery (5), internal medicine (3), pediatrics (3), obstetrics and gynecology (2), and 1 each from nephrology, neurosurgery, radiology, dermatology and ENT. Their clinical experience ranged from 8 to 32 years. Mean experience was 17 years. There were 5 Professors, 5 Associate professors, 7 Assistant professors and one clinical specialist without an academic title.

Semi-structured interview technique was chosen to gain a comprehensive understanding of participants’ views and to allow for exploration of vague answers. The interview questions aimed to find the clinicians understanding about altruism based on their clinical experiences, and the altruistic practices they considered as essential and others which they considered as optional. Interview questions were refined after conducting a pilot interview.

Interviews were conducted by MS, SI and SQ, all of whom are involved in planning and development of professionalism content in undergraduate medical curriculum, are experienced in teaching undergraduate medical students and involved in conducting communication skill sessions. This gave them a background understanding of altruism and helped in obtaining information from interviewees.

Each interview lasted between twenty to thirty minutes. Interviews were held through Zoom [11] with participants’ permission, audio-recorded and transcribed verbatim using Otter [12], to provide interactive transcripts in real time. Participant confidentiality was ensured.

Data saturation was assessed as no new information being generated from the last interviews. Transcribed data was checked for errors and participants were sent copies of their individual interview transcripts for review.

### Data analysis

Exploratory analysis of the data collected through clinicians’ responses was done. Two authors (MS and SQ) independently coded the transcripts using a qualitative data analysis and research software ‘Atlas ti- 8’. Open coding was done initially. Selective coding was then done by finding commonalities between codes to generate sub-themes and themes.

## Results

Regarding the interviewee characteristics, the ages of the participants ranged from 35 to 64 years. Mean age was 47 years. All participants were Muslim by religion. All were married, having children and some were living in joint family systems.

The main themes under which the research questions were explored were: ‘Clinicians’ understanding of altruism’, and ‘Practice points for altruism’, each having further subthemes identified from interview transcripts.

### A. Clinicians’ understanding of altruism

Almost all the participants recognized altruism as an important component of professionalism. Although the participants’ understanding of altruism was sought in the clinical context, we discovered a strong religious influence as well as a sociocultural impact. This is exemplified by the following quotes.

*“I think altruism is something inculcated in us not just professionally, but also culturally and especially religiously.” (interviewee 11, Female)*.

*“We should provide financial help to the poor patients because we are Muslims and we don’t consider it a burden or stress”. (interviewee 14, Male)*

*‘It is difficult to decide limits but being doctors and Muslims, we should always be ready for altruism.’ (interviewee 17, Male)*.

Role models were also reckoned as a powerful inspiration for few participants in our study, one of whom stated their impact as:

*“I’ve worked with amazing people that have shown altruism day in and out”. (interviewee11, female)*

The various concepts emerging from the interview transcripts regarding the clinicians’ understanding of altruism are listed below as subthemes, in descending order of frequency. The patient quotes relevant to the context are given in italics.

### 1. Prioritizing patient interest

Prioritizing patients above family commitments and own interests, emerged as the most repeated concept.

“*I think, to be a true professional. We should be keeping the patient’s interests at the priority, rather than our own interests.*” (Interviewee 3, male).

“*There was a time my daughter was on a wheelchair with suspected cerebral malaria and I was doing rounds in the hospital*” (Interviewee 11, female).

### 2. Making arrangements according to context/ situation

During the interviews, the participants were presented a hypothetical scenario where they faced a family emergency whilst having a patient/work related commitment and asked what they would do if such a situation occurred. This question was aimed to probe clinicians’ individual perceptions and understanding of altruism. Most of the participants said that given a situation where there was a clash between work and family commitment, they would request a colleague to manage the work commitment and most emphasized that they would weigh the context and nature of the issue and act accordingly.

“*I will analyze the situation and communicate with my family if my presence is beneficial for my mother, I will ask for another Doctor to replace me, …*” (Interviewee 6, female).

A few also stated that good patient communication and counselling in this regard helped in decision making.

*‘My approach is to counsel my patient and attendants. I have to set priority…… Patients really understand if properly communicated.’ (Interviewee 2, female)*.

### 3. Sacrificing extra time for patients

Many of the participants considered sacrificing their personal time beyond their duty hours, as altruism.

*“A patient I operated on was shifted to the ward for post op care, even though not necessary I came to see him multiple times during my free time and off days, even in the evenings….”* (Interviewee 8, female).

### 4. Physical exertion beyond obligation

This ranged from sacrificing rest or sleep to donating blood for the patient.

*“Altruism is when doctor gets up in the middle of night in the winter and attend the patient and especially in covid 19 scenario using full PPE in humid weather and knowing the risk.”* (Interviewee 5, male).

“*I donated blood twice for a young boy who was in shock after a roadside accident and matching blood group was not available.*” (Interviewee 18, female).

### 5. Providing financial help to patient

Participants also considered helping poor patients through provision of money, food or medicine.

*“I and many of my colleagues have arranged medicines …….we used to arrange for some zakat funds. And then we used to arrange their medicines.” (Interviewee 9, female)*.

### 6. Selflessness and empathy

Selflessness was talked about, in association with empathy for the patients by some participants and they explained it as:

*“Altruism is selfless empathy for patient betterment.”(Interviewee 4, female)*.

*“Empathy is a feeling, altruism is when you actually act.”* (Interviewee 11, female).

### 7. Favouring patients

 Few participants communicated their understanding of altruism as ‘favoring’ or guiding the patients beyond their usual duty.

*“It means if I can favour a patient beyond my routine work this is my understanding that this is altruism.”* (Interviewee 1, male).

### 8. Importance of teamwork in establishing altruism

The primary concept that emerged especially in a hospital based or team setting was the importance of teamwork in establishing altruism for patient benefit rather than an individual effort.

“*Medical profession is a teamwork and when you work in an institution there are always competent people around you*----” (Interviewee 4, female).

*“….and we must learn how to delegate work to other team members.”* (Interviewee 17, male).

### 9. Tailoring treatment according to patient needs and constraints rather than denying it altogether

An interesting concept suggested by one participant as a part of altruism was doing everything possible for the non-affording patients so that they are benefitted in the best possible way.

*“….we will offer them an alternative instead of denying them the procedure altogether. So basically, catering the patients according to their social structure and financial status*.” (Interviewee 8, female).

### 10. Positive psychological benefits

Some of the participants in our study reported a sense of satisfaction and divine connection with such altruistic acts:

*“Sometimes we become exhausted both mentally and physically when we work beyond our capacity but at the end we feel inner satisfaction.”***(**Interviewee 17, male).

*“… when we sacrifice our needs for others especially for the sick human beings then it is always rewarded by the Divine power’. (Interviewee 18, female)*

### 11. Compromise in work-life balance

While providing their understanding of altruism few of the participants recognized the work-life imbalance issues associated with being altruistic.

*“Sometimes you become exhausted and it becomes difficult for you to keep a balance between professional and family life.”* (Interviewee 14, male).

*“Neurosurgery is a subspecialty which requires a lot of sacrifice not only from doctor, but also from family as well….”* (Interviewee 16, female).

### 12. Relation with physical, mental or financial stress

Some participants recognized altruism having strong relation with stress.

*“In my opinion it is not possible to practice altruism without mental or physical stress.”* (Interviewee 18, female).

### 13. Difficult to practice altruism in essence

Few participants were of the understanding that altruism was difficult to practice if taken in its true meaning especially in an individual capacity.

*“So, in my personal opinion, it will be very difficult to incorporate altruism for practice. …. and there is a certain extent to which we can incorporate altruism into our practice.”* (Interviewee 3, male).

*“Since the system does not provide any resources for instance, transport or finance, it is not feasible for a single doctor to go out of their way to accommodate their patients.”* (Interviewee 8, female).

### B. Essential practice points for altruism

When posed a question, *“To what extent altruism should be an essential part of a doctor’s role as a professional and what practices do you consider as essential?”*, multiple concepts emerged from the answers. The participant comments related to essential practice requirements of altruism in workplace settings, were classified as subthemes and are presented in Table 1.

**Table 1:** Essential practice points of altruism and supportive quotes

Essential practice points	Interviewee quotes
Find a balance between altruistic tendency and work-life balance	“*I believe that keeping a balance between one’s altruistic tendencies and professional obligations is a must to provide long-term services to the patients.” (*Interviewee 10, female) *“….at the same time, there must be fine balance, one shouldn’t get so involved that it starts to affect you personally….”* (Interviewee 11, female)
Help patients according to individual capacity	*“what I think about it, and how our doctor can practice that depends upon each doctor as every doctor has his own capacity ….”.* (Interviewee *9*, female*)*
Train doctors consciously	“…. *it is something that requires more practical examples……… one can only fully learn what it means after experiencing it in everyday life with the right models*.” (Interviewee 8, female)
Establish Teamwork	*Team is beneficial. Whenever you have to face emergency you can call for help and can be handled easily.”* (Interviewee 2, female)
Generate funds	“I*f a patient can’t afford an investigation or a medication so to say, we have a ward fund to help in such a case.*” (11, female) *“We should generate funds for poor patients to reduce individual financial burden.”* (Interviewee 17, male)
Make decisions according to context	“….*can be decided only on case to case basis considering the patient’s expectations, doctor’s responsibilities, ….*” (Interviewee 12, male)
Facilitate patients	“*A lot of the times, all we can do is offer guidance to the patient, for instance if a patient requires blood ….all we can do is guide them”*. (Interviewee 8, female)
Communicate with patients	*“Patients really understand if properly communicated and have a professional approach.” (Interviewee 2*, female*)*
Be Empathetic	*“There is another element of ‘empathy’ in my opinion which is required. We should always think of ourselves in their shoes…”* *(*Interviewee *2*, female*)*
Realize limitations	*“But there are certain limits and, there is a certain extent to which we can incorporate altruism into our practice.”* *(*Interviewee *3*, male*)*
Be mentally prepared for stress	*“But when we choose this profession we are mentally prepared to face such situations like some mental and physical exertion and financial burden.”* (Interviewee 14, male)
The role of team leader	“*It is the attitude and sentiments of the head of institute, department and individuals to make environment suitable for altruism…*:” (Interviewee 5, male)
Enforced by the system rather than be an individual responsibility	*“…secondly, altruism should be enforced by the system and shouldn’t be your personal responsibility …”* (Interviewee 8, female)

Few participants commented on components of altruism which they deemed as optional or desirable rather than essential. The optional components with the salient relevant quotes were:

- Giving financial help to patients.

*“Giving financial help to patients is beyond the scope of professional altruism.”* (Interviewee 4, female).

- Working beyond duty hours.

*“If doctor A has finished his work timings and doctor B is available to take care of a patient, then doctor A may excuse to provide care to the patient so as not to violate the timing and responsibility of another doctor.”* (Interviewee 11, female).

- Doing non-designated work.

*“Similarly, a doctor can excuse from doing something for a patient if it is not his designated job….”* (Interviewee 7, female).

- Excusing from patient treatment when there was availability of a more competent doctor was also considered altruistic as it was in a patient’s best interest.

“*A doctor can excuse from doing something for a patient…. other professionals can do it better or more befitting to the job. Under these circumstances the moral consciousness of a doctor dictates him to the right decision for a patient….” (Interviewee 12, male)*.

## Discussion

Our study re-evaluates the practical understanding of altruism. It also suggests a realistic approach for students and physicians to deliver a more achievable and effective altruistic care which is sustainable. We used an exploratory, qualitative, interview-based approach to identify clinicians’ understanding of altruism based on their clinical experience and altruistic approaches they considered essential in various clinical contexts.

 For this purpose, our participants were from various specialties and age groups from both genders, having a background of institutional clinical practice and experience of teaching medical students. We found an influence of sociocultural and religious values on their perception regarding altruism and in their placement of altruism as an essential component of professionalism. The sociocultural norms and religious values of our region give high regard to helping others in need. According to Szuster, altruistic inspirations have an association with moral values [13]. As religious teachings stress upon moral values [9], altruism has a strong link to religion [14]. Research on regional differences in altruistic tendencies have also identified religion as one of the most important influencing factor [15, 16] as has culture [9]. Role modelling has been suggested significant in transmitting such sociocultural values [17]. This was an influencing factor for few participants in our study as well. There were no definite gender differences in participant opinions about altruism, although few studies have described females as being more altruistic [14].

We discovered that the understanding of clinicians regarding altruism was distinctly diverse. Opinions about it ranged from recognizing its positive psychological impact at one extreme, to it being considered as stressful and difficult to comply with on the other. Most of the participants considered altruism as prioritizing the patient’s interest above their own. However, when compelled to tackle the dual goals of practicing altruism and maintaining their work-life balance, they emphasized upon making decisions according to the context. Most recognized the sacrifice of time, money and effort for a patient, as important components of altruism, but some admitted to these components as stressful, rendering practice of altruism in its true essence impossible. This was also emphasized in other studies [6] with suggestions to change this practice for the well-being of both the physicians and the patients. More significantly, they agreed that empathizing with the patients and guiding them beyond the requirements of duty were more important and achievable aspects of altruism. An interesting finding was recognition of positive psychological benefits of such acts by some of the participants in our study. This appreciation of positive psychological benefits for altruism, was formerly taken as an ‘egoist’ or selfish motive [3, 4, 7]. There was an identified need for a balanced approach to altruism in some other studies [18] culminating in the more recent concept of ‘enlightened self-interest’ in altruism [8] which has a more balanced approach recognizing physician’s well-being as an advantage rather than a drawback. It promotes well-being of both the physicians and the patients as desirable [6].

Few new and interesting concepts emerged from the interviews. One of them was employing teamwork as an important means to establish altruism. Only a few studies have been identified linking effective altruism to team-work in an organizational setting [19] and none in the medical context. The importance of team work is doing the best for the patient in a situation where the onus of responsibility is on the team and not an individual. Therefore, even if one of the team members is not available or is committed elsewhere, the patient's interest is not compromised. However, this idea requires team organization and commitment [19]. The other concept was tailoring treatment according to patients’ needs and constraints and guiding them to alternate treatment options if they could not afford the primary treatment option, rather than denying treatment altogether. The essence of this idea was to do everything in one’s power to serve patients’ needs.

Moving on to the second theme, regarding the essential practice suggestions given by the clinicians, despite the variability in their concept of altruism they seemed to agree in most of their practical tips. The participants suggested a more realistic approach to practice altruism in the present-day clinical context. ‘Maintaining a balance’ between one’s altruistic tendencies, family life and mental health was considered as a top of the list essential for practice. This was followed by a related opinion of ‘identifying one’s own individual capacity’ to practice altruism as it varied according to the clinicians. Consistent with our findings, many studies also favoured the importance of a balanced approach between family life and professional commitments [20] encouraging development of ‘ethical literacy’ in students in order to respond appropriately in different contexts [4].

Another interesting suggestion was that of establishing ‘teamwork’ for an organized altruistic approach for improved patient care as well as doctors’ well-being. Although generating funds for needy patients was considered an essential practice component in our study, but by most as an organized team contribution in the form of ‘ward funds’, rather than an individual effort, for a long-term benefit. In a study by Babiker et al. [21] team based altruism was considered instrumental for feasible, consistent and sustainable patient care. The net effect of such an approach would not only benefit the patients but also promote the satisfaction and professionalism of doctors. Team leader was perceived as having a pivotal role in such organized efforts. Jahan et al. regarded organizational contribution as an important factor [22]. Health care institutes can provide better patient care by implementing a team-based philosophy [21].

The clinicians in our study also placed more emphasis on small day to day, achievable altruistic activities in the spirit of empathizing with patients and guiding them, with their interest in mind. In a study by Sanjai & Gopichandran [17], these acts were defined as acts of ‘Simple altruism’ where there wasn’t a high level of physical, mental, social or emotional risk to oneself. Whereas acts like blood donation or working in risky environments were taken as ‘risk-taking altruism’. There has been considerable evidence supporting the empathy-altruism hypothesis suggesting empathy as a motivator for altruism [3, 23]. Jeffrey has also suggested a practical model of empathy focusing on developing skills, attitudes and moral concern [23]. It was asserted by the participants that formal training of doctors in this context was essential mainly through an experiential model. It has been traditionally considered that the ‘altruistic approach’ implied giving financial help to patients, working beyond duty hours and doing non-designated work [24], as can also be deciphered from the examples narrated by the participants. However, our results suggest that clinicians considered these components as ‘optional’ for practice based on an individual’s moral consciousness rather than an obligation. This has also been suggested in other studies [3, 17].

### Implications

The results from this study have important implications, at the conceptual and practical level for both medical education and medical practice.

For the medical educationists and curriculum developers, the findings of this study may help to reduce the ambiguity in developing professionalism component of medical curricula, for medical students of both preclinical and clinical years. At the conceptual level, altruism may be re-defined to fit the intricacies of modern-day society. Revisions in curricula, with more emphasis on empathy towards patients, better communication skills, better team-work and practices which are feasible for the doctors and are in patients’ interest should be the focus. At the practical level, the study results provide a framework for the essential expected behaviours especially regarding student teaching. Students may be taught the essential altruistic behaviours in a more practical and meaningful manner, by developing case-based scenarios in pre-clinical years and workplace-based techniques in the clinical years.

The results also have vital importance for medical practice especially in Medical institutions and hospitals. They can guide the clinicians to develop altruistic attitudes and behaviours which benefit their patients and are feasible as well as sustainable. They can also guide the administration to promote a supportive institutional culture enabling and encouraging clinicians towards altruistic practices and teamwork.

### Limitations and future recommendations

Our study was done on purposely selected sample of 18 clinicians, thus it may not be possible to generalize the findings widely. The participants were from teaching institutions with hospital-based practices and their perspectives may pertain to team situations rather than individual practice. The context was kept limited, as the focus of the research was identifying essential altruistic behaviours to teach medical students, as part of professionalism. Further studies are suggested to identify essential altruistic practices and behaviors in other contexts such as individual-based and private practices where lack of teamwork and economic pressures are some of the constraints.

## Conclusions

Altruism is an important professionalism attribute for doctors but is a complex concept subject to variations in medical practice and physician backgrounds. In this study, we identified a wide variation in clinicians understanding of altruism and shared suggestions for establishing effective and realistic altruism in medical practice. These include a balanced altruistic approach and promotion of workplace altruistic cultures through organized team-based approach rather than individual efforts.

## Supplementary Information

**Additional file 1:** Interview guide.

## Data Availability

The datasets generated and/or analyzed during the current study are not available publicly so as not to compromise participant anonymity. However, they are available from the corresponding author on reasonable request.
