# Supplementary material for: ‘The best interest of patients, not self-interest’: how clinicians understand altruism
Source: BMC Med Educ. 2021 Sep 7;21:477. doi: 10.1186/s12909-021-02908-0 (PMC8421461; doi:10.1186/s12909-021-02908-0)
Supplement: Supplementary file 1 — Additional file 1: Interview guide. [file 12909_2021_2908_MOESM1_ESM.docx]

| Interview objectives | Main Questions | Supplementary questions when required |
| --- | --- | --- |
| Questions related to in-depth understanding of altruism in clinicians | What do you understand about the term ‘altruism’? | How do you consider it as a part of professionalism? |
|  | Given a scenario that you have planned to do a procedure tomorrow (bronchoscopy/ gastroscopy/cardiac catheter/ planned surgery) and your father has been rushed into a hospital in another city for an emergency surgery. Your mother would like you to be with her during this difficult time. What will you do? |  |
|  | Can you please narrate a clinical experience in your practice where you think you practiced altruism? |  |
| Questions related to practice points for altruism | In your opinion, what are the essential practice components of altruism without which a doctor cannot be called a professional? | In your opinion is there an extent or limit to altruism that should be an essential part of a doctor’s role as a professional. |

Additional file 2: Interview guide
